# Comprehensive analysis of transcriptome response to salinity stress in the halophytic turf grass *Sporobolus virginicus*

**DOI:** 10.3389/fpls.2015.00241

**Published:** 2015-04-21

**Authors:** Naoki Yamamoto, Tomoyuki Takano, Keisuke Tanaka, Taichiro Ishige, Shin Terashima, Chisato Endo, Takamitsu Kurusu, Shunsuke Yajima, Kentaro Yano, Yuichi Tada

**Affiliations:** ^1^Bioinformatics Laboratory, Department of Life Sciences, School of Agriculture, Meiji UniversityTama-ku, Kawasaki, Japan; ^2^NODAI Genome Research Center, Tokyo University of AgricultureSetagaya-ku, Japan; ^3^School of Bioscience and Biotechnology, Tokyo University of TechnologyHachioji, Japan; ^4^Department of Bioscience, Tokyo University of AgricultureSetagaya-ku, Japan

**Keywords:** halophyte, transcriptome, *Sporobolus virginicus*, turf grass, next-generation sequencing, salt stress, osmotic adaptation, ion exclusion

## Abstract

The turf grass *Sporobolus virginicus* is halophyte and has high salinity tolerance. To investigate the molecular basis of its remarkable tolerance, we performed Illumina high-throughput RNA sequencing on roots and shoots of a *S. virginicus* genotype under normal and saline conditions. The 130 million short reads were assembled into 444,242 unigenes. A comparative analysis of the transcriptome with rice and Arabidopsis transcriptome revealed six turf grass-specific unigenes encoding transcription factors. Interestingly, all of them showed root specific expression and five of them encode bZIP type transcription factors. Another remarkable transcriptional feature of *S. virginicus* was activation of specific pathways under salinity stress. Pathway enrichment analysis suggested transcriptional activation of amino acid, pyruvate, and phospholipid metabolism. Up-regulation of several unigenes, previously shown to respond to salt stress in other halophytes was also observed. Gene Ontology enrichment analysis revealed that unigenes assigned as proteins in response to water stress, such as dehydrin and aquaporin, and transporters such as cation, amino acid, and citrate transporters, and H^+^-ATPase, were up-regulated in both shoots and roots under salinity. A correspondence analysis of the enriched pathways in turf grass cells, but not in rice cells, revealed two groups of unigenes similarly up-regulated in the turf grass in response to salt stress; one of the groups, showing excessive up-regulation under salinity, included unigenes homologos to salinity responsive genes in other halophytes. Thus, the present study identified candidate genes involved in salt tolerance of *S. virginicus*. This genetic resource should be valuable for understanding the mechanisms underlying high salt tolerance in *S. virginicus*. This information can also provide insight into salt tolerance in other halophytes.

## Introduction

Halophytes are recognized as plants that survive under high salt concentrations that cause other plants to die (Flowers and Colmer, 2008). Le Houérou (1993) mentioned that there are 5000–6000 halophytic species in the world. Broad diversity in salt tolerance abilities in halophytes have been reported, with the tolerable salt concentration ranging from 200 to 1500 mM (Flowers and Colmer, 2008; Tada et al., 2014). Due to the need to cultivate marginal lands to increase food production, elucidating salt tolerance mechanisms in halophytes is important. Identification of key genetic information related to this ability could lead to expanding vegetation to high-salinity environments such as the sea and deserts.

Two main factors, osmotic and ionic stress, negatively affect plant growth under high-salinity conditions (Maathuis et al., 2014). Growth is reduced by salinity via distinct processes in the osmotic phase and the ionic phase (Munns and Tester, 2008; Shavrukov, 2013; Roy et al., 2014). These phases can be experimentally distinguished by measuring effects in the short term (within minutes to a few days) upon addition of salt or after longer times (several days to weeks). Proposed mechanisms of salt tolerance in halophytes are based on several concepts, such as ion homeostasis, compartmentalization, exclusion and secretion of ions, and transport (Liphschtz and Waisel, 1974; Flowers et al., 1977; Teakle and Tyerman, 2010; Deinlein et al., 2014; Gupta and Huang, 2014; Roy et al., 2014). Accumulation of low-molecular mass compounds termed compatible solutes or osmolytes is also documented as an important biochemical mechanism in both halophytes and glycophytes for coping with the high osmolarity of salt (Flowers et al., 1977; Ashrafa and Foolad, 2007). In addition, several reports suggest that salt tolerance in plants can be partially explained by the scavenging properties of antioxidative proteins (Abogadallah, 2010; Bose et al., 2014). Although much knowledge of mechanisms common to halophytes has accumulated, a complete understanding of the specific molecular mechanisms underlying salt tolerance in individual halophytes is lacking.

The C_4_ turf grass *Sporobolus virginicus*, which is found in tropical to warm temperate regions, is one of the well-documented halophytes (Blits and Gallagher, 1991; Marcum and Murdoch, 1992). Due to its high level of salinity tolerance, great efforts have been made to elucidate its mechanism. *S. virginicus* excludes excess salt from roots and secretes it from salt glands on leaf surfaces (Naidoo and Naidoo, 1998a,b). For osmotic adjustment, *S. virginicus* accumulates compatible solutes such as glycinebetaine, proline and sugars depending on salinity (Marcum and Murdoch, 1992). Recently, we isolated a genotype of *S. virginicus* from a seashore in Japan to characterize its remarkable tolerance of salinity up to 1.5 M NaCl (Tada et al., 2014). Compared to other genotypes in the USA, South Africa and Egypt, the Japanese genotype represented higher salt tolerance (Tada et al., 2014). The Japanese genotype shows a distinctive relationship between regulation of Na^+^, Cl^−^, and K^+^ influx/efflux and proline accumulation during growth of both whole plants and cultured cells (Tada et al., 2014). Monitoring of biological events at the molecular level may offer a deeper understanding of the salinity tolerance mechanism of this genotype.

In the present study, we aimed to identify genetic information involved in salinity tolerance of the Japanese genotype of *S. virginicus*. Comparative transcriptome analysis is a powerful approach for discovery of the molecular basis behind particular biological events (Roy et al., 2011; Ward et al., 2012; Halimaa et al., 2014). This approach not only allows us to mine change in gene expression under specific conditions, but also to detect unique transcripts in the organisms (Dhaubhadel et al., 2007; Wei et al., 2010; Huan et al., 2013). Dang et al. (2013) applied this approach to a halophyte *Reaumuria trigyna* and observed up-regulation of genes related to ion transport and the reactive oxygen species scavenging system. Sun et al. (2013) found up-regulation of genes involved in stress tolerance, signal transduction, energy production and conversion, and inorganic ion transport in a halophytic forage grass.

Here, we examined the comprehensive transcriptome response of the Japanese genotype of *S. virginicus* under saline and non-saline conditions using Illumina next-generation sequencing. Also, comparison of the transcriptome response in *S. virginicus* with that in rice under salinity was conducted to screen the global response at the metabolic pathway level underlying the salinity tolerance of *S. virginicus*. Our results identified candidate gene sequences involved in biosynthesis of compatible solutes and in exclusion and secretion of salts. In addition, we identified salt-responsive transcription factors, which are specific to the halophyte. To the best of our knowledge, this is the first report to provide molecular insights into the salinity tolerance of *S. virginicus*.

## Materials and methods

### Plant materials

Rhizomes of the Japanese genotype of *S. virginicus*, collected at Iriomote Island, Okinawa, Japan (Tada et al., 2014), were cultivated in disposable culture pots filled with vermiculite at 27°C with a 12 h light/12 h dark cycle under 350 μmol photons m^−2^ s^−1^ of fluorescent light. The Home Hyponica 501 hydroponics device (Kyowa Co. Ltd., Osaka, Japan) was used for culturing. Turf grass was vegetatively propagated by cuttings in culture solution. After 4 weeks, the plants were transplanted to fresh hydroponic culture solution with or without 500 mM NaCl. Plants in each plot were separated into shoots and roots. At 48 h after transplanting, the plants were frozen in liquid nitrogen and stored at −80°C until use.

### Transcriptome sequencing

Each plant sample was powdered using a mortar and pestle in liquid nitrogen, and total RNA was extracted from the powder using an RNeasy Plant Mini Kit (Qiagen, Hilden, Germany). Quality and concentration of RNA were tested by an RNA 6000 nano kit (Agilent, Santa Clara, CA) on a 2100 Bioanalyzer (Agilent). Total RNA fractions with a concentration of 0.1–4.0 μg and RNA integrity ≥8 were used for further experiments.

Each RNA fraction was used to prepare a separate cDNA library for sequencing using a TruSeq RNA Sample Prep Kit v2 (Illumina, Inc., San Diego, CA) following the manual. In brief, poly(A) RNAs were selected, fragmented, and annealed with random primers to synthesize first-strand cDNAs. After conversion to double-stranded cDNAs, the termini were repaired to blunt the ends and add adenine to the 3′ termini. The cDNAs were then ligated to index adapters (AR006, AR005, AR012, and AR019) provided by Illumina. Subsequently, the cDNAs were amplified by PCR with a set of primers corresponding to the cDNA termini. The amplified cDNAs were purified using Agencourt AMPure XP beads (Beckman Coulter Genomics, Danvers, MA) to eliminate small cDNAs. The amount and quality of the resultant cDNA fraction was estimated using a DNA 1000 kit (Agilent) on a 2100 Bioanalyzer (Agilent). The concentration of cDNA was determined more accurately by qRT-PCR using a KAPA Library Quantification Kit (Kapa Biosystems, Wilmington, MA).

Each cDNA library was employed for in-house sequencing on the HiSeq 2500 sequencing system (Illumina) with 100 bp paired-end reads. Raw data were converted into fastaq format. The data were deposited to the DDBJ Sequencing Read Archive database (accession number DRA002969).

### Assembly of transcriptome short reads

Raw sequence data were pre-processed to extract high-quality reads for unigene (transcriptome contig) construction according to the following procedures. First, any redundant pairs of reads were deleted to minimize calculation complexity and eliminate adapter sequences and poly(A) and poly(T) sequences by cutadapt software (Martin, 2011). Next, to exclude tRNA and rRNA sequences, BLAST searches of the reads were carried out against tRNA and rRNA sequences in rice (RAP-DB, Sakai et al., 2013) and Arabidopsis (TAIR, Lamesch et al., 2012) with an *e*-value of 1e-5. After eliminating probable tRNA/rRNA sequences, low-quality sequences [quality value (QV) < 10] at both ends of the reads were trimmed. The resultant reads were screened using the following four criteria: (1) average QV ≤ 17; (2) length ≤ 20 bp; (3) low-quality nucleotide sequences (QV < 10) <10% of the total; and (4) the sequence contained no ambiguous “N's.”

All the pre-processed reads from the four samples were assembled into unigenes. We tested three short read assembly pipelines, Trinity ver. 2013-02-25 (Grabherr et al., 2011), Velvet ver. 1.2.10 (Zerbino and Birney, 2008; Zerbino et al., 2009)/Oases ver. 1.2.10 (Schulz et al., 2012), and SOAPdenovo-Trans ver.1.03 (Xie et al., 2014). For Velvet, we used the optional parameter “-ins_length 200.” In Oases, we used the parameter “-ins_length2 200 –scaffolding yes.” In SOAPdenovo-Trans, we used the parameter “rd_len_cutof = 100, avg_ins = 200, reverse_seq = 0, asm_flags = 3, map_len = 32.” Different parameter settings were applied; namely, k-mer length and insert length in Velvet/Oases and SOAPdenovo-Trans to find an optimized assembly method. After evaluation of the assemblies, the resultant contigs from the optimized conditions were designated “unigenes.”

### Homologos sequence search

Unigene sequences were used to conduct searches against some publicly available sequence databases by the BLAST program (Altschul et al., 1990). The nucleotide database “nt” was searched by BLASTN with a threshold e-value of 1e-5. The non-redundant protein database “nr” and the universal protein knowledgebase “UniProtKB” were searched by BLASTX with the same criteria. The rice all protein databases of the MSU rice genome annotation project were also searched by BLASTX for mining of orthologous proteins (Kawahara et al., 2013). The Arabidopsis all protein database (TAIR10, Lamesch et al., 2012) was also searched by the BLASTX program.

To map the unigenes to metabolic pathways, a KEGG Orthology search was carried out on the KEGG Automatic Annotation Server by the BBH (bi-directional best hit) method against data of any species in the KEGG database (Mao et al., 2005; Kanehisa et al., 2014).

For further annotation of unigenes showing significant homology with proteins in the UniProtKB, we conducted an InterProScan search to assign protein domains and Gene Ontology (GO) terms (Zdobnov and Apweiler, 2001).

### Mining of differentially expressed genes

Raw sequencing data were pre-processed by the same procedures used in unigene construction as described in the section “Assembly of transcriptome short reads” without deletion of redundant pairs of reads. The pre-processed reads were mapped to the unigenes constructed in this study using the Burrows–Wheeler Aligner (Li and Durbin, 2009) with the default parameter settings. Differentially expressed unigenes were identified by comparison between the with and without 500 mM NaCl experimental plots in shoots and roots, respectively, by Kal's *Z*-test with Bonferroni adjustments of *P*-values (Bland and Altman, 1995). Unigenes were designated as differentially expressed when the corrected *P*-value was ≤0.05.

### Gene set enrichment analysis

Gene set enrichment analyses (Wu and Lin, 2009) of GO terms and KEGG metabolic pathways were conducted on each set of differentially expressed unigenes. The set of unigenes expressed in each organ was used as a reference for the analysis. Enriched GO terms or KEGG metabolic pathways were statistically analyzed by Fisher's exact test and the false discovery rate of Bonferroni adjustments (Fisher, 1922).

### Mining of salt-responsive genes in rice

We used a massively parallel signature sequencing data set of leaves and roots from rice under normal and saline conditions (Shen et al., 2011; GSM629213, GSM629218, GSM629210, and GSM629215) from the NCBI Sequence Reads Archive (http://www.ncbi.nlm.nih.gov/sra). The raw data were processed to exclude adapter sequences, and low-quality sequences were eliminated at the following thresholds: (1) average QV ≤ 17; (2) length ≤16 bp; (3) low-quality nucleotide sequences (QV < 10) <10% of the total; and (4) the sequence contained no ambiguous “N's.” The resultant sequences were mapped to coding sequences of the rice all gene model of the Rice Genome Annotation Project (Kawahara et al., 2013). Genes differentially expressed in leaves and roots were surveyed using the same criteria described in the section on “Mining of differentially expressed genes” above.

### Correspondence analysis

For correspondence analysis of gene expression of all unigenes, expression levels using Fragments Per Kilobase of transcript per Million mapped (FPKM) values for each unigene were calculated using an in-house Perl script (Howe et al., 2011). The expression data were analyzed as described by Yano et al. (2006).

### Real-time qRT-PCR

Expression of 7 unigenes differentially expressed under salinity was analyzed by real-time qRT-PCR. The names of the unigenes and primer sequences are listed in Supplementary Table 1. Relative expression of the selected unigenes in shoots and roots treated with 500 mM NaCl for 48 h were calculated. First-strand cDNA was synthesized from 500 ng of total RNA using a QuantiTect Reverse Transcription Kit (Qiagen) according to the manufacturer's instructions. The cDNA fraction was diluted 40-fold, and 1 or 4 μL was used for real-time qRT-PCR. qRT-PCR was carried out using QuantiNova SYBR Green PCR Kit (Qiagen) or QuantiFast SYBR Green PCR Kit (Qiagen) on the Eco Real-Time PCR System (Illumina). Relative expression of each target gene to the actin gene was calculated by the delta-delta Ct method.

## Results

### Transcriptome sequencing

In our previous study, the Japanese genotype of *S. virginicus* showed stagnated shoot growth and stimulated root growth under over 500 mM of NaCl (Tada et al., 2014). To characterize transcriptome response in the organs separately, we prepared the following four sample plots: “shoot-control” (CS, shoots under normal conditions); “shoot-NaCl” (NS, shoots under salinity condition by NaCl); “root-control” (CR, roots under normal conditions); and “root-NaCl” (NR, roots under salinity condition by NaCl). Hydroponically grown plants under normal conditions for 4 weeks were conducted to 500 mM of NaCl treatment for NS and NR, or 0 mM of NaCl treatment at the same time for CS and CR (mock plots). We previously observed that turf grass starts accumulating Na^+^ and Cl^−^ within a few days of 500 mM NaCl treatment (Tada et al., 2014). To analyze transcriptional events in the phase, we sampled the shoots and roots at 48 h after the start of salt stress treatment in this study.

Transcriptome sequencing using the HiSeq 2500 system resulted in generating approximately 29–48 million pairs of short reads for each experimental plot. For constructing a set of unigenes (transcriptome contigs), the raw reads were pre-processed by the four steps described in Materials and Methods (Supplementary Table 2). The total number of the resulting pair of high-quality reads was 28–44 million. For identification of differentially expressed unigenes, the raw reads were pre-processed without elimination of redundant sequences. The resultant short reads used for gene expression analysis were 47, 36, 31, and 29 million reads in the CS, NS, CR, and NR plots, respectively.

### *De novo* assembly

**Figure 1:**
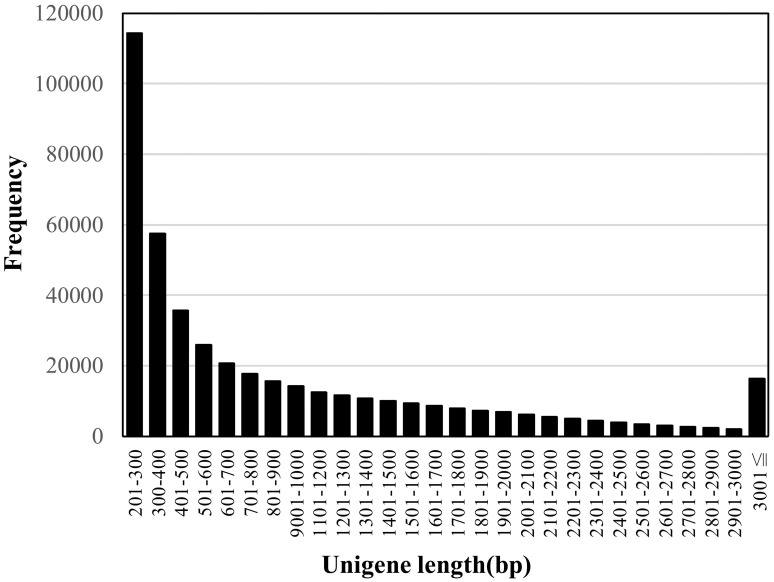
**Size distribution of unigenes**.

In order to generate unigenes under the optimal assembly conditions, we tested three transcript assembly pipelines (Trinity, Velvet/Oases, and SOAPdenovo-Trans). The longest N50 length of 1518 was obtained in Trinity, comparing with those of 1211 and 1775 in SOAPdenovo-Trans and Velvet/Oases, respectively. When the sets of transcriptome contigs were searched against the rice proteome by BLASTX, we obtained higher coverage in the rice proteome when using Trinity (45.7%) than using Velvet/Oases (43.7%). Therefore, we employed the Trinity assembly in this study. A summary of the assembly statistics is shown in Table 1. The 444,242 contigs obtained were designated “unigenes” to avoid confusion in terminology. The size distribution of the unigenes is shown in Figure 1. The unigenes were used to conduct a search against the nucleotide database “nt” in NCBI by the BLASTN program. In total, 208,728 unigenes showed similarities to sequences in the database with statistical significance (1e-5). Half of the sequences hit (104,152) were from a C_4_ plant foxtail millet. The species corresponding to the second and third most frequent hits were also C_4_ plants sorghum and maize, with 28,708 and 12,687 hits, respectively.

**Table 1:** **Summary of transcript assembly**.

Total reads	270,558,254
Total unigenes	444,242
Size (Mb)	403.9
Minimum transcript length (bp)	201
Maximum transcript length (bp)	15272
Average transcript length (bp)	909.1
N50 length (bp)	1518
GC content (%)	47.8

### Functional annotation of unigenes

Homology-based functional annotation of the unigenes was carried out. The unigene sequences were searched against the universal protein knowledgebase (UniProtKB) (Apweiler et al., 2004) and the non-redundant protein database (nr) using the BLAST program. As a result, 22.9% of the unigenes (101,700) showed statistically significant (*e*-value of 1e-5, 50% coverage) homology to proteins in the UniProtKB database (Table 2). Among the homologos sequences in UniProtKB, 31,614 were independent. For the nr database, 38.5% of the unigenes (248,862) showed homology under the same criteria (Table 3). Among the homologos sequences in nr, 93,643 were independent. Even when the threshold was set as an e-value of 1e-5, 44% of unigenes were unassigned by the BLASTX search, presumably because most of them were too short (data not shown).

**Table 2:** **Summary of functional annotation of unigenes**.

**Database**	**Unigenes having homologos sequence**		**Unigenes having homologos sequence (≥50% coverage)**	
	**Number**	**Hit (%)**	**Number**	**Hit (%)**
nt	208,728	47.0	68,480	15.4
Uniprot	167,971	37.8	101,700	22.9
nr	248,862	56.0	171,003	38.5
rice	227,726	51.3	137,365	30.9
Arabidopsis	194,652	43.8	112,247	25.3
KEGG	21,133	4.8	–	–
Any^*^	280,342	63.1	255,207	57.4

* *Unigenes having at least one hit sequence in any database listed*.

**Table 3:** **Differentially expressed transcription factors**.

	**Unigene ID**	**Family**	**ID of homologos transcription factor**	**Species**	***E*-value in BLASTX**	**Shoot**			**Root**		
						**Gene expression level**		***P*-value corrected by Bonferroni**	**Gene expression level**		***P*-value corrected by Bonferroni**
						**CS**	**NS**		**CR**	**NR**	
Up-regulated	comp164617_c0_seql	bZIP	Ghi016560	*Gossypium hirsutum*	3.0E-95	6.3E-08	0	1	2.1E-05	5.8E-05	2.4E-38
	comp288664_c0_seq1	bZIP	PK07387.1	*Cannabis sativa*	4.0E-08	–	–	–	3.1E-06	1.5E-05	8.6E-16
	comp383711_c0_seq1	bZIP	Pta007702	*Pinus taeda*	1.0E-38	–	–	–	1.2E-06	7.3E-06	4.3E-07
Down-regulated	comp337275_c0_seq1	C3H	Bna025760	*Brassica napus*	5.0E-06	–	–	–	5.8E-06	0	6.9E-10
	comp97693_c0_seq2	bZIP	evm_27.model. AmTr_v1.0_scaffold00081.79	*Amborella trichopoda*	3.0E-07	–	–	–	4.8E-06	0	2.0E-07
	comp487069_c0_seq1	bZIP	Ghi016560	*Gossypium hirsutum*	1.0E-79	–	–	–	5.3E-06	1.9E-07	2.0E-07

To give GO terms and protein domain information for the unigenes, an InterProScan search was conducted. Out of 167,971 unigenes having homologos sequences in UniProt (*e*-value of 1e-5), 91,125 (54.3%) were assigned GO terms as follows: 58,049 in Biological Process, 82,865 in Molecular Function and 24,797 in Cellular Component. The distribution of GO slim terms, which gives a broad overview of GO content, is shown in Supplementary Figure 1. A few GO slim terms occupied a large portion (approximately 90%) of the total in all cases. This distribution was very similar to that in rice (Supplementary Figure 1). The InterProScan search categorized 1737 unigenes into the GO slim term “response to stimulus” [GO:0050896], which includes the narrow-sense term “response to stress” [GO:0006950]. The GO term “response to stress” was assigned to 395 unigenes, which contained such unigenes encoding dehydrin, universal stress protein, and abscisic acid stress ripening protein. At least one protein domain was predicted for each of 110,370 of the unigenes. In total, 9380 kinds of protein domains were identified (Supplementary Table 3). We found stress-related protein domains, including water stress, osmotic stress and oxidative stress-related domains, predicted in the unigenes. The universal stress protein A domain was present at the highest frequency (100 unigenes compared to others with no more than 14).

**Figure 2:**
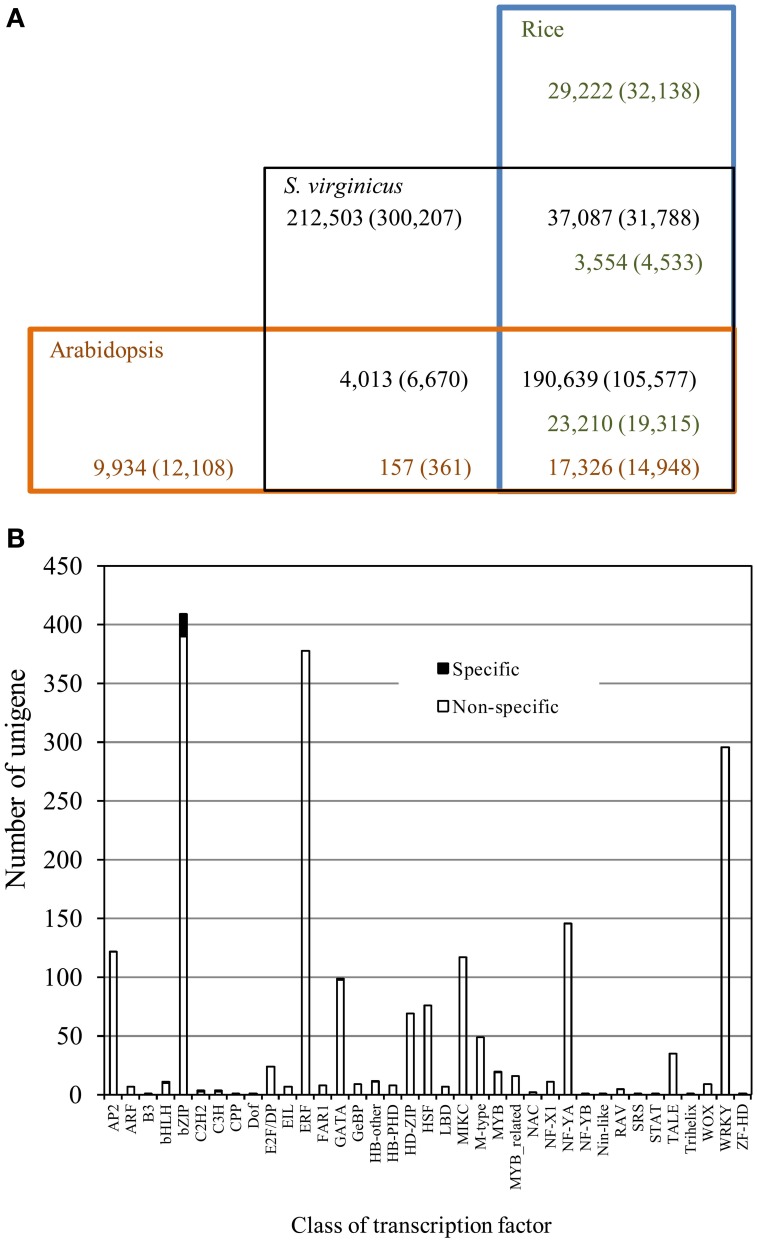
**Conservation and divergence of *S. virginicus* unigenes with the rice and Arabidopsis proteomes. (A)** Number of unigenes in *S. virginicus* is shown in black. Number of proteins in rice and Arabidopsis are shown in green and red, respectively. The number at different BLAST search thresholds (1e-5, 50% coverage) is represented in parentheses. **(B)** Distribution of transcription factors in *S. virginicus*. Black and white bars represent number of transcription factor family members in *S. virginicus*-specific unigenes and that of non-specific unigenes, respectively.

To compare the gene repertoire of turf grass to glycophytes, we conducted BLASTX searches of the unigenes against both the rice and Arabidopsis all protein databases. As shown in Table 2 and Figure 2A, approximately 51.3% of the translated unigenes were statistically significant (1e-5), similar to protein sequences in rice. In the search, 26,814 protein sequences (47.9% of all the proteins) were assigned to the unigenes. For Arabidopsis, 17,483 protein sequences (43.8%) were assigned to the unigenes with statistical significance. Approximately 45% of the translated unigenes in turf grass showed significant homology with both rice and Arabidopsis proteins. Except for unigenes common to all of the 3 plant species, between turf grass and rice, 3554 (8.3%) of the translated unigenes showed significant (1e-5) homology with rice proteins. In contrast, only 157 (7.4%) of the translated unigenes showed significant homology with Arabidopsis proteins. Approximately 40% of unigenes were categorized as turf grass-specific transcripts. Among the unigenes, we found 25 transcription factor unigenes by a BLASTX search against proteins in the Plant Transcription Factor Database (Jin et al., 2014) (Supplementary Table 4). Interestingly, we observed biased distribution of turf grass-specific unigenes to some transcription factor families (Figure 2B). In particular, unigenes encoding bZIP were clearly over-represented in the unigenes (Supplementary Table 4).

In order to predict unigenes involved in metabolic pathways, we performed a KEGG Orthology (KO) search (Mao et al., 2005; Kanehisa et al., 2014). As a result, 15,296 unigenes were assigned with at least one KO identifier (KO ID), and 77.5% of all KO IDs were covered by the unigenes (Supplementary Table 6). These unigenes were clustered into 4708 and 4209 gene models in rice and Arabidopsis, respectively. In rice and Arabidopsis, 7304 and 4654 gene models were respectively assigned in the KEGG database. By integrating the KO search results with the earlier BLAST results, we assigned functional annotations to 280,342 unigenes (255,207 at the threshold of 50% coverage) (Table 2). The complete gene annotation list is provided in Supplementary Table 8.

### Differential gene expression analysis

**Figure 3:**
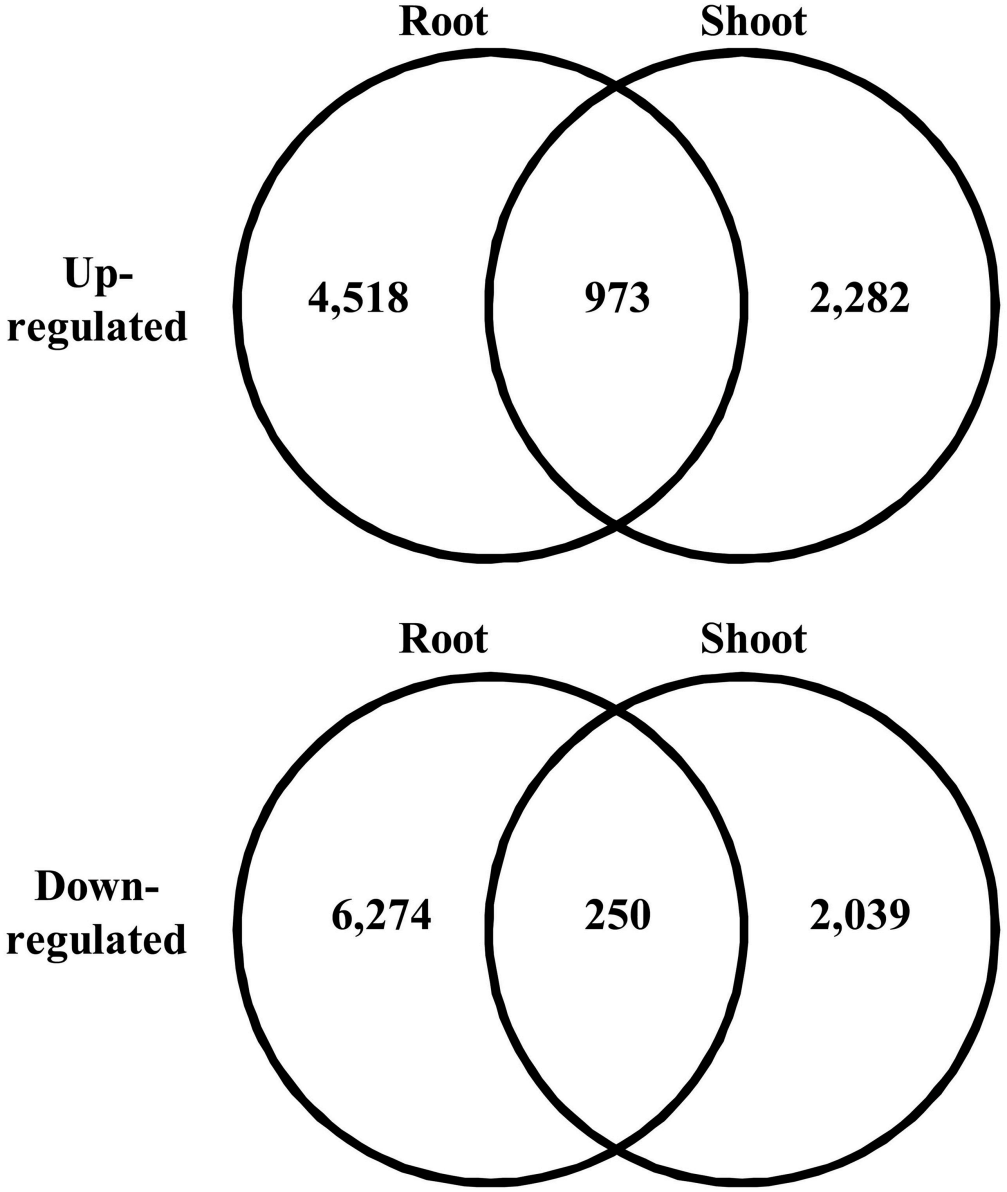
**Venn diagram of differentially expressed unigenes under high salt conditions**. The number of unigenes in each category is given in each circle.

We searched for differentially expressed unigenes that responded to salinity. Among all the short reads, we employed only short reads that mapped to a single unigene to avoid overestimation of gene expression at low levels. In total, 31.9–37.6% of short reads mapped to unigenes as a single hit. We observed expression of 187,344 and 270,226 unigenes in shoots and roots, respectively. Salinity-responsive unigenes were searched by comparing counts of mapped short reads between control and salt-treated samples using Kal's *Z*-test with the Bonferroni correction. Consequently, 2.0 and 1.7% of all the unigenes represented up-regulation in roots and shoots, respectively, with 5% statistical significance (Figure 3). Among the unigenes up-regulated in roots, 973 were up-regulated in shoots. Among the unigenes down-regulated in roots, 250 were down-regulated in shoots. These unigenes might be involved in fundamental biological responses to salt stress in turf grass cells.

We checked gene expression of 25 unigenes encoding transcription factors that were not found in the glycophytes rice and Arabidopsis. Notably, expression of 22 of these unigenes were detected only in roots. Furthermore, 6 unigenes (bZIP and C3H) showed differential expression in roots (Table 3).

### Go enrichment analysis

Gene set enrichment analyzes of GO terms was conducted to comprehensively interpret gene expression data for up- and down-regulated unigenes (Supplementary Table 7). The number of enriched GO terms in the up-regulated unigenes was 59 in shoots and 125 in roots, and the number in the down-regulated unigenes was 88 in shoots and 138 in roots.

GO terms enriched in up-regulated unigenes in both shoots and roots contained such as “response to stress” [GO:0006950] and “response to water” [GO:009415] (Supplementary Tables 7A,B). The categories include up-regulated unigenes encoding dehydrin, universal stress protein, abscisic stress-ripening protein, heat shock protein and late embryogenesis abundant protein (Supplementary Table 5A). We also found enrichment of GO terms for ion transport-related categories, including “transport” [GO:0006810] and “transmembrane transport” [GO:0055085] (Supplementary Table 5B). In roots, 81 unigenes in these categories showed up-regulated expression. Some of these unigenes were assigned the GO terms “ATPase activity, coupled to transmembrane movement of substances” [GO:0042626], “ATPase activity” [GO:0016887], “amino acid transmembrane transporter activity” [GO:0015171], and “cation transport” [GO:0006812] (Supplementary Table 5B). The categories related to ATPase included 8 unigenes encoding ABC transporters, which transport Cl^−^. In the category of “amino acid transmembrane transporter activity,” 8 unigenes encoding amino acid permease, which plays a role in transport of amino acids were included. Among them one unigene (comp392924_c0_seq1) encodes a proline-specific permease. In the category of “cation transport,” a unigene for high-affinity potassium transporter, HKT, (comp127987_c0_seq1) was included. In shoots, the category of “transport” and “transmembrane transport” were overrepresented, which contained 44 unigenes for such as ABC transporter, aquaporin, polyol/monosaccharide transporter, and sugar/inositol transporter.

GO terms enriched in down-regulated unigenes in shoots included categories related to photosynthesis (GO:0015979, GO:0009765, GO:0015995). In roots, GO categories related to protein synthesis, modification and degradation (GO:0006412, GO:0006508, GO:0015031, GO:0006457) were identified.

### Pathway enrichment analysis

To inspect induction of biological events at the metabolic pathway level under salinity, a KEGG pathway enrichment test was carried out. In shoots and roots, 17 and 58 KEGG pathways, respectively, were up-regulated, including “Arginine and proline metabolism” [ko00330] and “Phosphonate and phosphinate metabolism” [ko00440] (Supplementary Table 9). In “Arginine and proline metabolism,” unigenes encoding delta-1-pyrroline-5-carboxylate synthase (P5CS, EC 1.2.1.41) and N-acetyl-glutamate synthase (EC 2.3.1.1) were found. The unigenes in “Arginine and proline metabolism” also included genes for the polyamine biosynthetic pathway: arginine decarboxylase (EC 4.1.1.19), polyamine oxidase (EC1.5.3.14), S-adenosylmethionine decarboxylase (EC 4.1.1.50) and spermidine synthase (EC 2.5.1.22). Under “Phosphonate and phosphinate metabolism,” unigenes encoding aminoalcoholphosphotransferase (AAPT, EC 2.7.8.1), ethanolamine-phosphate cytidylyltransferase (ECT, EC 2.7.7.14), and CTP: phosphorylcholine cytidylyltransferase (CCT, EC 2.7.7.15) were up-regulated. AAPT catalyzes reactions from diacylglycerol and a CDP-aminoalcohol to phosphatidylcholine and phosphatidylethanolamine. ECT catalyzes a reversible reaction from CTP and ethanolamine phosphate to diphosphate and CDP-ethanolamine. CCT catalyzes the reversible conversion from CTP and choline phosphate to diphosphate and CDP-choline.

Notably, 16 of the up-regulated pathways in shoots also showed enrichment in roots (Table 4A). Most of them were primary metabolic pathways. Approximately 60% of the up-regulated unigenes categorized into these pathways were commonly observed in both sets of samples (data not shown). In particular, unigenes in “Biosynthesis of amino acids,” “Carbon metabolism,” “Oxidative phosphorylation,” and “2-Oxocarboxylic acid metabolism” were shared at a high proportion (82.1, 87.5, 81.3, and 75.0%, respectively).

Table 4**KEGG metabolic pathways enriched in differentially expressed unigenes**.

**Table:** 

**(A) *S. virginicus* IN BOTH SHOOTS AND ROOTS**								
**KO ID**	**KO description**		**Number of unigene assigned by KO**		***P*-value corrected by Bonferroni**	**Number of unigene assigned by KO**		***P*-value corrected by Bonferroni**
			**Shoot**	**Reference**		**Root**	**Reference**	
ko01100	metabolic pathways		147/3255 (4.52)	2093/187938 (1.11)	4.2e-43	503/5491 (9.16)	4255/270724 (1.57)	3.9e-223
ko01110	biosynthesis of secondary metabolites		65/3255 (2.00)	1108/187938 (0.59)	1.4e-14	294/5491 (5.35)	2080/270724 (0.77)	1.8e-150
ko01230	biosynthesis of amino acids		28/3255 (0.86)	326/187938 (0.17)	1.8e-09	108/5491 (1.97)	671/270724 (0.25)	1.1e-61
ko00330	arginine and proline metabolism		15/3255 (0.46)	90/187938 (0.05)	1.3e-08	36/5491 (0.66)	234/270724 (0.09)	6.2e-21
ko01120	microbial metabolism in diverse environments		30/3255 (0.92)	416/187938 (0.22)	2.5.e-08	158/5491 (2.88)	955/270724 (0.35)	5.9.e-89
ko01200	carbon metabolism		24/3255 (0.74)	293/187938 (0.16)	1.4e-07	121/5491 (2.20)	709/270724 (0.26)	6.5e-72
ko00564	glycerophospholipid metabolism		13/3255 (0.40)	98/187938 (0.05)	4.4e-06	14/5491 (0.25)	144/270724 (0.05)	1.6e-06
ko00052	galactose metabolism		11/3255 (0.34)	80/187938 (0.04)	3.8e-05	29/5491 (0.53)	158/270724 (0.06)	2.5e-19
ko00270	cysteine and methionine metabolism		12/3255 (0.37)	105/187938 (0.06)	8.3e-05	33/5491 (0.60)	229/270724 (0.08)	2.0e-18
ko00190	oxidative phosphorylation		16/3255 (0.49)	196/187938 (0.10)	1.1e-04	76/5491 (1.38)	425/270724 (0.16)	3.0e-47
ko00630	glyoxylate and dicarboxylate metabolism		8/3255 (0.25)	65/187938 (0.03)	4.4e-03	27/5491 (0.49)	184/270724 (0.07)	1.5e-15
ko00260	glycine, serine and threonine metabolism		9/3255 (0.28)	90/187938 (0.05)	7.3e-03	34/5491 (0.62)	197/270724 (0.07)	1.7e-21
ko01210	2-oxocarboxylic acid metabolism		8/3255 (0.25)	73/187938 (0.04)	1.0e-02	38/5491 (0.69)	185/270724 (0.07)	1.0e-26
ko00620	pyruvate metabolism		9/3255 (0.28)	103/187938 (0.05)	2.1e-02	45/5491 (0.82)	241/270724 (0.09)	1.8e-29
ko00565	ether lipid metabolism		5/3255 (0.15)	28/187938 (0.01)	2.9e-02	7/5491 (0.13)	57/270724 (0.02)	1.5e-04
ko00520	amino sugar and nucleotide sugar metabolism		10/3255 (0.31)	139/187938 (0.07)	4.4e-02	39/5491 (0.71)	277/270724 (0.10)	4.0e-21

**Table:** 

**(B) ONLY IN *S. virginicus***								
**Change**	**KO ID**	**KO description**	
Up-regulated	ko00052	Galactose metabolism	
	ko00190	Oxidative phosphorylation	
	ko00270	Cysteine and methionine metabolism	
	ko00564	Glycerophospholipid metabolism	
	ko00565	Ether lipid metabolism	
	ko00620	Pyruvate metabolism	
	ko01120	Microbial metabolism in diverse environments	
Down-regulated	ko00010	Glycolysis/Gluconeogenesis	
	ko00020	Citrate cycle (TCA cycle)	
	ko00030	Pentose phosphate pathway	
	ko00051	Fructose and mannose metabolism	
	ko00053	Ascorbate and aldarate metabolism	
	ko00071	Fatty acid degradation	
	ko00250	Alanine, aspartate, and glutamate metabolism	
	ko00260	Glycine, serine and threonine metabolism	
	ko00270	Cysteine and methionine metabolism	
	ko00330	Arginine and proline metabolism	
	ko00350	Tyrosine metabolism	
	ko00380	Tryptophan metabolism	
	ko00450	Selenocompound metabolism	
	ko00520	Amino sugar and nucleotide sugar metabolism	
	ko00620	Pyruvate metabolism	
	ko00680	Methane metabolism	
	ko00720	Carbon fixation pathways in prokaryotes	
	ko00910	Nitrogen metabolism	
	ko00980	Metabolism of xenobiotics by cytochrome P450	
	ko00982	Drug metabolism—cytochrome P450	
	ko01120	Microbial metabolism in diverse environments	
	ko01212	Fatty acid metabolism	
	ko01230	Biosynthesis of amino acids	

### Comparison of transcriptome response to that in rice under salinity

To roughly extract distinguishable transcripts in halophytes, we compared the transcriptome data to that of a glycophyte, rice. For comparison, we used massively parallel signature sequencing data from rice treated with 250 mM NaCl for 24 h (Shen et al., 2011). When we mapped the reads to rice all transcript sequences, approximately 72–74% of short reads were associated with transcripts of a single locus. Differentially expressed genes in leaves and roots were separately screened for statistical significance (5% level), resulting in extraction of 1076 and 980 genes, respectively (Supplementary Table 10).

**Figure 4:**
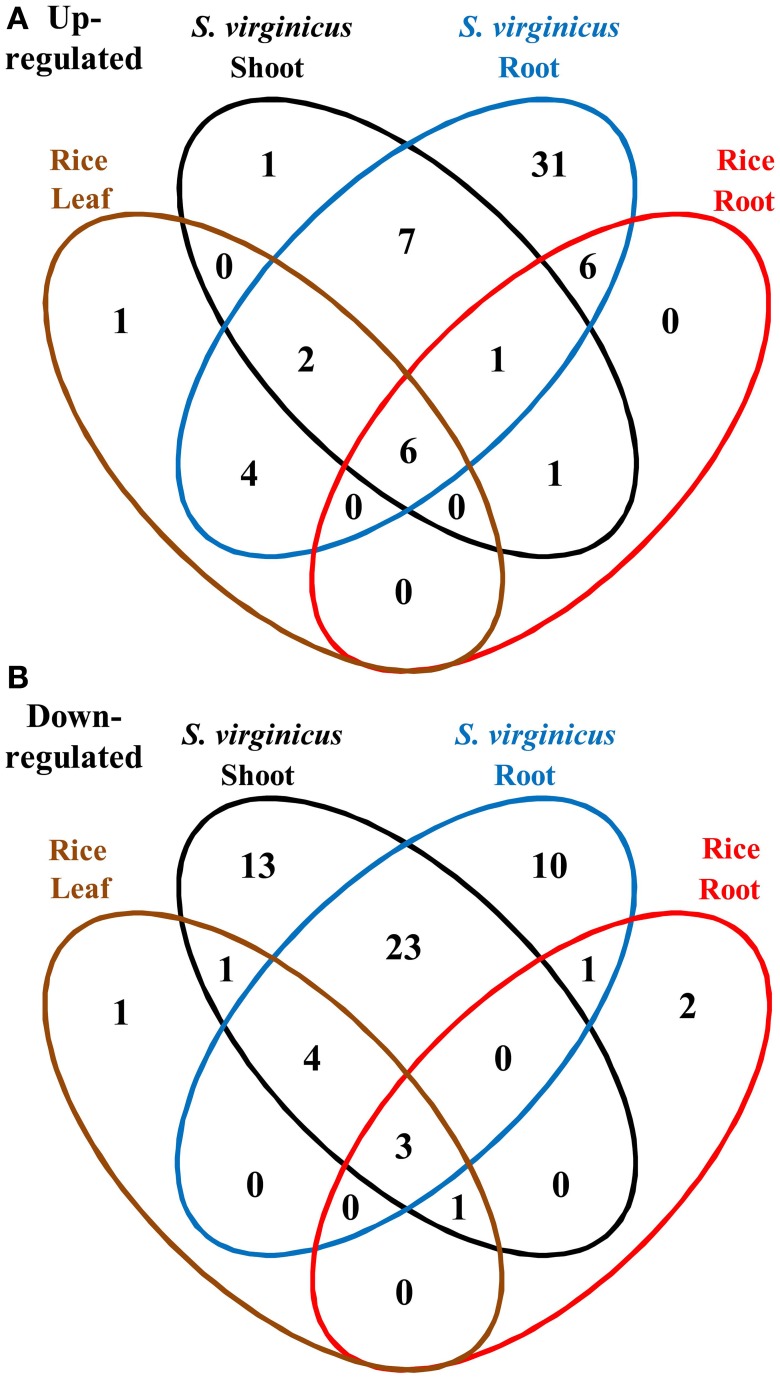
**Venn diagram of metabolic pathways enriched among differentially expressed unigenes under salinity. (A)** Up- and **(B)** down-regulated pathways. Fifteen categories are represented by ellipses in different colors. Black: shoots of *S. virginicus*, blue: roots of *S. virginicus*, brown: leaves of rice, and red: roots of rice. The number of metabolic pathways in each category is shown.

To compare the global transcriptome response in metabolic pathways of turf grass and rice, pathway enrichment analysis of the differentially expressed genes in rice was carried out. Enrichment was observed in 28 pathways (Supplementary Table 11). Combining the results with those of turf grass, enriched pathways were categorized in a Venn diagram (Figure 4, Supplementary Table 12). Between turf grass and rice, 39 up-regulated and 46 down-regulated pathways were detected as turf grass-specific. The 39 up-regulated pathways included “Glycerophospholipid metabolism” [ko00564], “Ether lipid metabolism” [ko00565], and “Pyruvate metabolism” [ko00620]. Among the turf-grass specific pathways responding to salinity, 7 were up-regulated and 23 were down-regulated in both shoots and roots (Table 4B). On the other hand, 6 pathways, including “Carbon metabolism” [ko01200], “Glycine, serine, and threonine metabolism” [ko00260], and “Arginine and proline metabolism” [ko00330], were up-regulated and 3 pathways were down-regulated in both species. These results are preliminary because experimental conditions for the two plant species were different.

### Correspondence analysis of up-regulated pathways specific to *S. virginicus*

**Figure 5:**
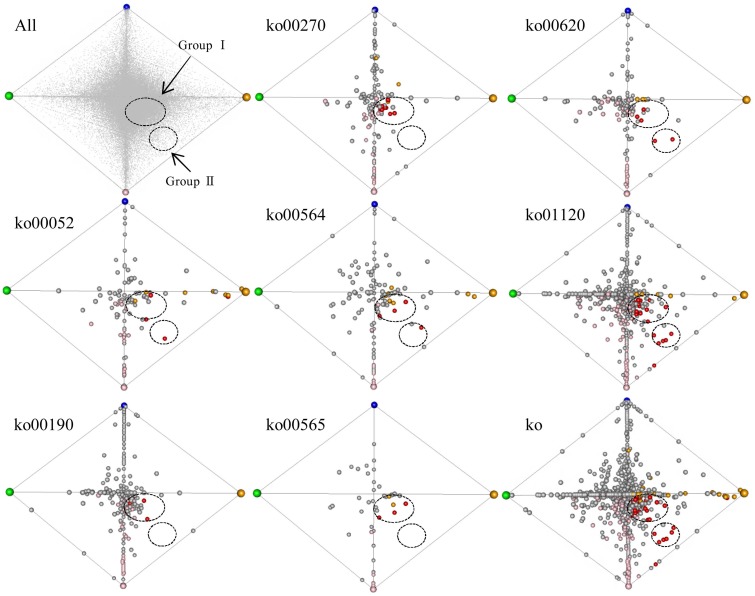
**Correspondence analysis plot of all unigenes**. Each unigene was plotted in a tetrahedron. The summit of the tetrahedron represents a guide gene showing a sample-specific gene expression pattern: CS (shoots under normal conditions) in green, CR (roots under normal conditions) in blue, NS (shoots under salinity condition by NaCl) in orange and NR (roots under salinity condition by NaCl) in pink. Upper left tetrahedron represents plots of all unigenes. Each unigene is shown in gray. The other 8 tetrahedrons represent plots of unigenes with KO annotations. The tetrahedron “ko” represents plots of unigenes annotated with any of the 7 KO annotations overrepresented in up-regulated unigenes in both organs. Each unigene is shown in a circle: unigenes up-regulated only in shoots are yellow, unigenes up-regulated only in roots are pink, and unigenes up-regulated in both organs are red. Clusters of unigenes are within dashed circles.

To survey co-regulated unigenes involved in the 7 enriched (up-regulated) pathways specific to turf grass, we applied a correspondence analysis (Yano et al., 2006) to the transcriptome data. The unigenes processed by correspondence analysis were plotted on a tetrahedron, in which the coordinate of unigenes was given according to their expression similarity (Figure 5). We observed that some of the unigenes assigned to pathways were located near the summits of the tetrahedron. This result suggests that the turf grass genome encodes salt-responsive genes specific to each organ. In addition, we observed two clusters of unigenes assigned with one or more of these 7 pathways: one cluster (designated group I) was located near the midsection of the tetrahedron, and another was located near an edge of the tetrahedron (designated group II). The results imply the existence of at least two gene groups within the up-regulated metabolic pathways that are similarly regulated in response to salt stress in turf grass.

Group I was comprised of unigenes showing minor fold-changes in expression in response to salt treatment in both shoots and roots, including 8 unigenes encoding ATPases (Supplementary Table 13). Group II consist of 6 unigenes showing a large change in gene expression level following salt treatment in both shoots and roots, including pyruvate dehydrogenase complex E1 alpha subunit (EC 1.2.4.1), NADP-dependent malic enzyme (EC 1.1.1.40), transketolase (EC 2.2.1.1), phosphofructokinase (PFK, EC 2.7.1.11) and phosphoethanolamine N-methyltransferase (PEAMT, EC 2.1.1.103) (Supplementary Table 13).

### Identification of differentially expressed unigenes

**Figure 6:**
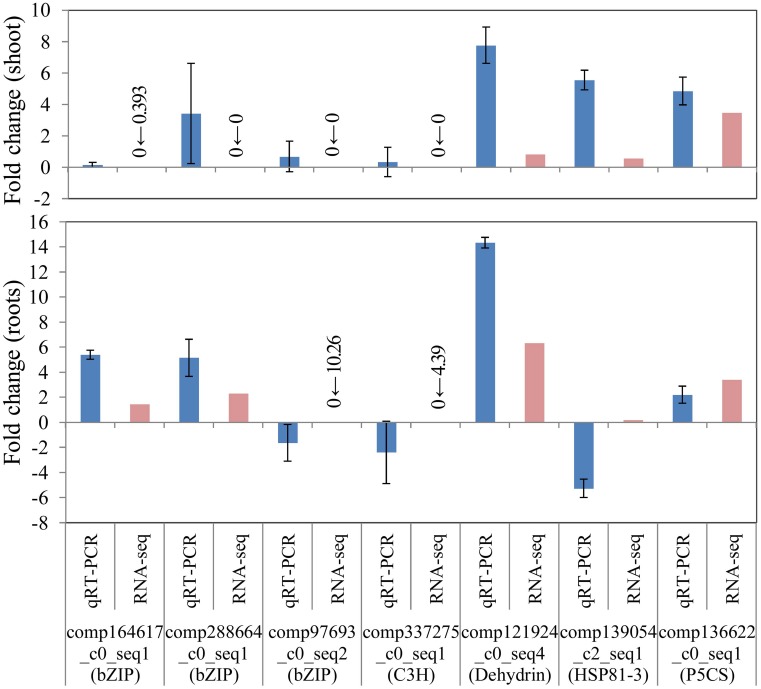
**Verification of RNA-seq results by real-time quantitative PCR (qRT-PCR)**. FPKM values were calculated from globally normalized RNA-seq data. Data of qRT-PCR are represented as means ± SE of three biological replicates. Figures before and after the arrows indicate FPKM values of samples under saline and normal conditions, respectively.

To confirm the differential gene expression of unigene candidates underlying salinity tolerance in *S. virginicus*, we performed real-time qRT-PCR. Nine differentially expressed unigenes were chosen: 4 unigenes for transcription factors in Table 3, a unigene for dehydrin, a unigene for heat shock protein 81-1 in Supplementary Table 5, and a unigene for P5CS. We observed the expected gene expression responses, similar to those found in the RNA sequencing results in most cases, except for *HSP81-3* (Figure 6). These results demonstrate the reliability of the differential gene expression analysis.

## Discussion

In the present study, we generated *S. virginicus* transcriptome sequencing data to reveal molecular factors underlying the salinity tolerance. The present study elucidated that regulation of genes for transcription factors, transporters, stress response proteins and enzymes involved in amino acid, pyruvate, and phospholipid metabolisms might be related to the salinity tolerance of *S. virginicus* (Table 4B).

It is remarkable that the transcriptome of *S. virginicus* included 19 genes for bZIPs that were not found in glycophytes, rice or Arabidopsis (Table 3). Interestingly, five of the *bZIP* genes showed root-specific salt-responsive expression. Overexpression of a bZIP from the woody halophyte *Tamarix hispida* increased tolerance to drought and salt in Arabidopsis (Ji et al., 2013). Liu et al. (2014) reported involvement of bZIP family proteins in plant responses to dehydration and salt stress. The transcription factor bZIP24 was suppressed in a halophytic Arabidopsis relative, *Lobularia maritima*, under salinity, resulted in activated expression of genes involved in cytoplasmic ion homeostasis and osmotic adjustment; the Na^+^ transporter AtHKT1; the Na^+^/H^+^ antiporter AtSOS1; the aquaporin AtPIP2.1; and a glutamine synthetase (Yang et al., 2009). In contrast, the counterpart in Arabidopsis was induced under salinity. Golldack et al. (2011) reviewed the involvement of several transcription factor families, including bZIP, AP2/ERF, MYB, NAC, WRKY and zinc finger proteins, in plant adaptation to saline conditions and presented the model that bZIP receives a signal of stress from MYB and regulates zinc finger proteins, MYB AP2/ERF, and NAC, which are located down-stream of the signaling pathway. Thus, bZIP might play a central role in conferring stress tolerance. Our results imply that the overrepresented bZIP proteins may play a specific role in the salinity response of turf grass. Further experimental tests of gene function or identification of target cis-elements will be required to identify the role of these five bZIPs in *S. virginicus*.

Under saline conditions, plants commonly accumulate compatible solutes, especially proline (Ashrafa and Foolad, 2007; Szabados and Savouré, 2010; Gupta and Huang, 2014). Accumulation of proline in *S. virginicus* is induced depending on the strength of salinity (Naidoo and Naidoo, 1998a). We have observed induction of proline synthesis in shoots, roots, and cultured cells of *S. virginicus* and much higher proline accumulation in cultured cells of *S. virginicus* than rice cultured cells under salinity (Tada et al., 2014). It was also reported that proline transporters play roles in salt tolerance (Rentsch et al., 1996). In this study, we observed transcriptional activation of *P5CS* in both shoots and roots, which catalyze the rate-limiting step of glutamate-derived proline biosynthesis (Strizhov et al., 1997). Expression of amino acid transporters in turf grass was also induced by salt treatment. In addition, activation of the 2-oxoacid biosynthetic pathway at the mRNA level was detected in both *S. virginicus* and rice. 2-oxoacids, such as pyruvate, oxaloacetate and α-ketoglutarate, are produced in glycolysis and the TCA cycle, and utilized for amino acid biosynthesis (O'Leary et al., 2011). The main initial products in amino acid biosynthesis from 2-oxoacids are asparagine and glutamate. Asparagine is synthesized from oxaloacetate by aspartate aminotransferase and glutamate from α-ketoglutarate by oxoglutarate aminotransferase (Miesak and Coruzzi, 2002; Hodges, 2002). These amino acids are then converted into various other amino acids (Forde and Lea, 2007; Jander and Joshi, 2009). Glutamate is consumed for proline and polyamine biosynthesis. Glutamine and glutamate could also be utilized as compatible solutes (Roberts, 2005). These compatible solutes would contribute to osmotic regulation of cells, though the contribution of other amino acids to salt tolerance is diverse among plant species and growth conditions (Mansour, 2000; El-Shintinawy and El-Shourbagy, 2001; Nasir et al., 2010; Hakim et al., 2014). Therefore, 2-oxoacid provision may be a rate-limiting factor in the biosynthesis of compatible solutes under high salinity. Fougère et al. (1991) reported a decrease in organic acid content with increase of amino acid content in alfalfa under salt stress. Therefore, we hypothesize that up-regulation of amino acid metabolism/synthesis including pyruvate metabolism under salinity stress might play a distinct role in the salinity tolerance mechanism. Garg et al. (2014) revealed key metabolic pathways, including amino acid biosynthesis, involved in stress tolerance in wild halophyte rice, *P. coarctata*.

Next-generation sequencing techniques have been applied to investigate the molecular basis of salinity tolerance in halophytes. Oh et al. (2010) reported that salt-inducible expression of *sodium/proton antiporter* (*SALT OVERLY SENSITIVE1*) (Shi et al., 2000) is a distinguishable feature of *Thellungiella* species from Arabidopsis. Genes for cation transport, abscisic acid signaling, and wax production in *R. trigyna* (Wu et al., 2012) and genes related to ion transport and the reactive oxygen species scavenging system in *T. salsuginea* (Dang et al., 2013) were reported to be possible contributors for their salt tolerant mechanisms. In *G. aridum*, pathways involved in “transport,” “response to hormone stimulus,” and “signaling” play important roles during salt stress, while genes involved in “protein kinase activity” and “transporter activity” undergo major changes in expression during early and later stages of salt stress, respectively (Xu et al., 2013). Sun et al. (2013) observed up-regulation of genes in categories including “inorganic ion transport” in *L. chinensis* under saline-alkaline stress. Thus, up-regulation of genes for transporter is commonly observed in these halophytes under salinity stress. In this study, we also found enrichment of GO terms for ion transport-related categories in *S. virginicus* under salinity stress, which include genes encoding cation transporters and H^+^-ATPase (Supplementary Tables 5B, 13). Compartmentalization, exclusion and secretion of Na^+^ are crucial for adaptation of glycophytes and halophytes under salinity (Apse et al., 1999; Shi et al., 2003; Flowers and Colmer, 2008; Teakle and Tyerman, 2010; Gupta and Huang, 2014; Roy et al., 2014). These transporters would play roles to transport excess ions to the vacuole or senescent tissues. In *S. virginicus*, exclusion of ions from roots and secretion of ions from salt glands primarily function in achievement of salt balance (Naidoo and Naidoo, 1998a). Unigenes encoding dehydrin and aquaporin, which were reported to be associated with salt gland (Tan et al., 2013; Jyothi-Prakash et al., 2014), were up-regulated under salinity (Supplementary Table 5A). Thus, genes products related to compartmentalization, exclusion and secretion of ions might play critical roles for salinity tolerance in *S. virginicus*.

In this study, correspondence analysis revealed two gene groups that were similarly regulated in response to salt stress in turf grass (Figure 5, Supplementary Table 13). Some of the group II gene products were suggested to have a relation to salinity tolerance in halophytes. Pyruvate dehydrogenase was reported as a salt-responsive protein in *Thellungiella halophila* (Wang et al., 2013). NADP-dependent malic enzyme was responsive to salt stress in *Mesembryanthemum crystallium* (Cushman, 1992). A proteomic analysis showed up-regulation of transketolase in the mangrove *Kandelia candel* under short-term salt stress (Wang et al., 2014), although down-regulation of transketolase was reported in *Halogeton glomeratus* (Wang et al., 2015). Suzuki et al. (2005) reported that PFK activity was increased in halophytic suspension-cultured cells from *Bruguiera sexangula* under salinity. A PEAMT in *Atriplex nummularia* L. was described as an enzyme involved in glycine betaine biosynthesis (Tabuchi et al., 2005). Thus, *S. virginicus* shared the common feature with other halophytes in point of regulation of these genes. The co-regulation of the group II genes could be key genetic factors for salinity tolerance in *S. virginicus*.

## Concluding remarks and future directions

**Figure 7:**
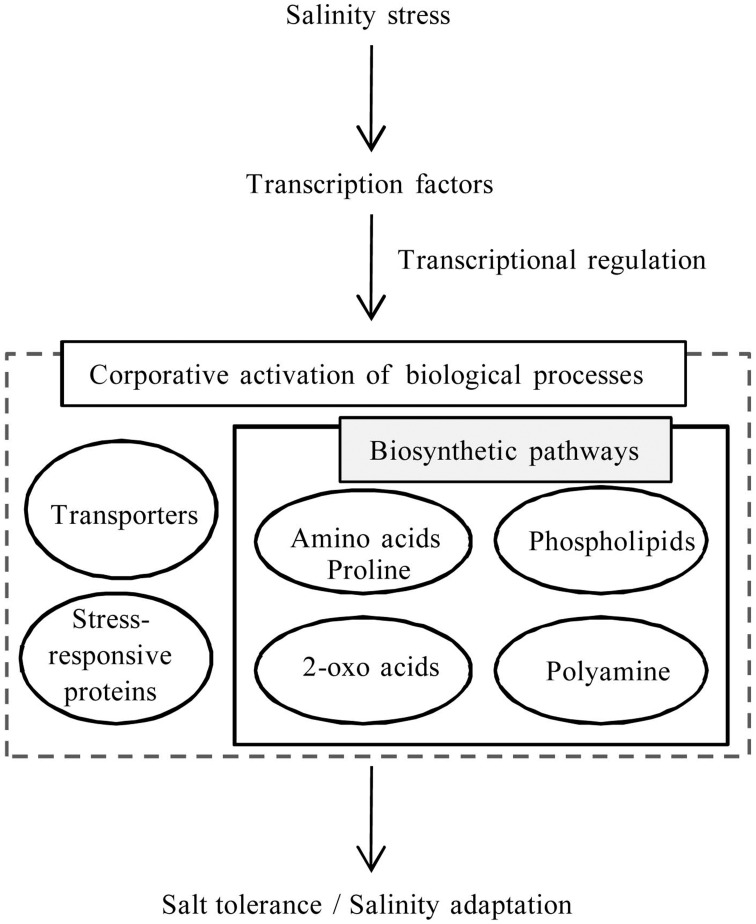
**Schematic model for expression of salinity tolerance and adaptability**.

Transcriptome analysis of *S. virginicus* revealed several unigene sequences to help address its molecular mechanism for salinity tolerance. By integrating the results, we proposed a model for the remarkable salt tolerance and adaptation to salinity in *S. virginicus* (Figure 7). Co-activation of several biological processes, such as transporters and amino acid, 2-oxoacids, and phospholipid metabolisms, is likely to constitute the biological response in *S. virginicus* under salinity. Our next goal is to determine the function of the key genes involved in each biological process related to the response under salinity. One direction is characterization of the transcription factor genes, *bZIP* and *C3H*, which might control the intracellular biological processes related to the salinity response. A strategy for ectopic expression of the transcription factor genes in transformed plants may accelerate identification of downstream gene candidates related to salinity tolerance in *S. virginicus* and promote further understanding of plant salinity tolerance. The transcriptome data should be a useful resource for better understanding of the mechanisms of salinity tolerance in plants.

### Conflict of interest statement

The authors declare that the research was conducted in the absence of any commercial or financial relationships that could be construed as a potential conflict of interest.

## Abbreviations

GO: gene ontology
KO: KEGG orthology.
