# Supplementary material for: Comprehensive analysis of transcriptome response to salinity stress in the halophytic turf grass Sporobolus virginicus
Source: Front Plant Sci. 2015 Apr 21;6:241. doi: 10.3389/fpls.2015.00241 (PMC4404951; doi:10.3389/fpls.2015.00241)
Supplement: Supplementary file 14 [file Image1.PDF]

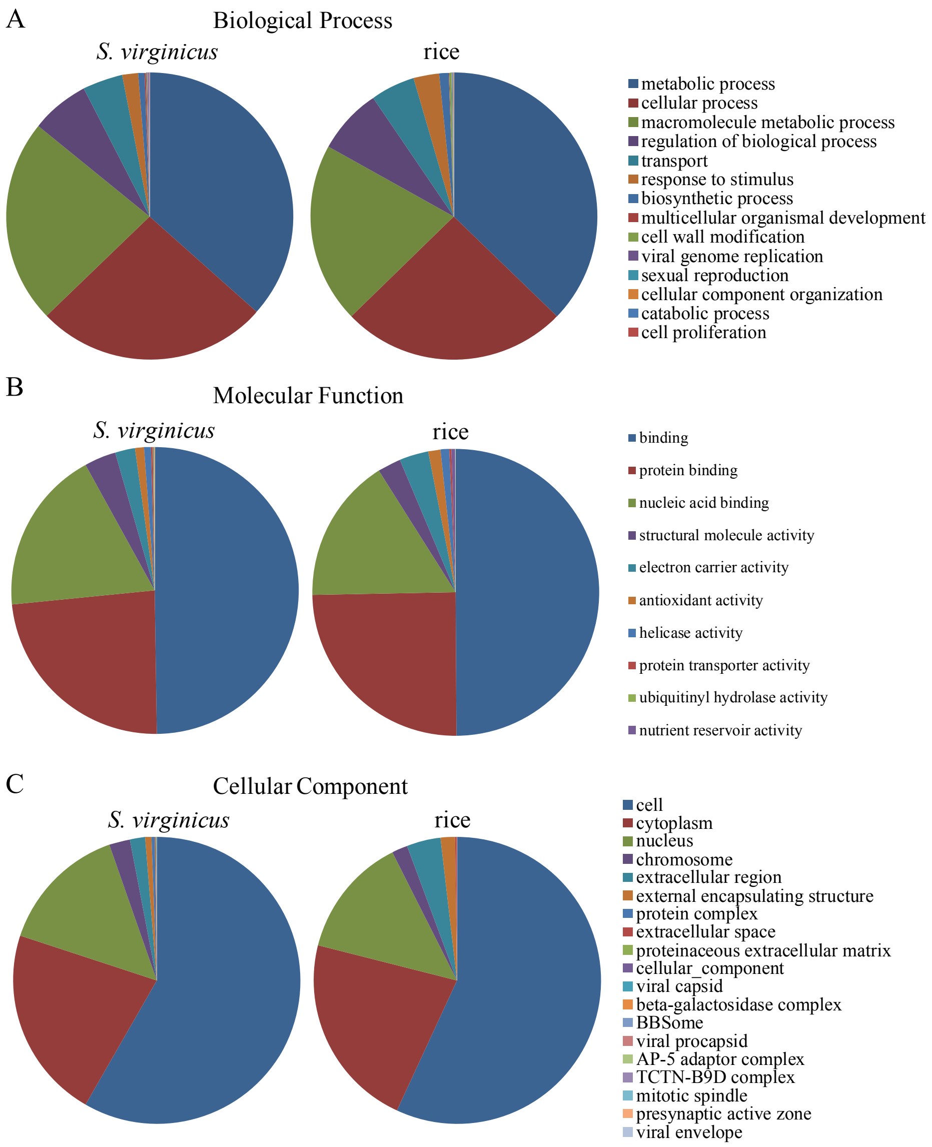


Supplementary Figure 1. Functional categorization of unigenes based on GO slim terms. The proportion of GO slim terms is shown for *S. virginicus* unigenes and the rice proteome. (A) Biological Process, (B) Molecular Function, (C) Cellular Component.
